# Working in Venezuela: How the Crisis has Affected the Labor Conditions

**DOI:** 10.29024/aogh.2325

**Published:** 2018-09-25

**Authors:** Yohama Caraballo-Arias, Jesús Madrid, Marcial C Barrios

**Affiliations:** 1 Central University of Venezuela (UCV), the Associate Venezuelan Group for the Iberoamerican Cochrane Network, Foundation Learning and Development on Occupational Health (LDOH), International Commission on Occupational Health, VE; 2 Universidad Católica Andrés Bello, VE; 3 Mundo Ocupacional C.A., VE

## Abstract

**Background:**

Venezuela, the country with the largest oil reserves in the world, is facing the worst economic, social and political crisis in its history; which has notably affected the quality of life of the workforce and the entire population.

**Objectives:**

Identify and analyze the main social factors derived from the Venezuelan crisis, which are affecting the workers’ health and working conditions.

**Methods:**

Document study. Several sources of information from the last twenty years were consulted, ranging from public statistics and reports, newspaper articles, and results of scientific research. The information gathered was carefully studied to ensure that only reliable sources were used to ultimately reach valid conclusions.

**Results:**

Both workers from the formal and informal sector and their families are struggling to fulfill their basic needs. Low salaries and soaring inflation have resulted in a dramatic reduction in the purchasing power of the people. General violence and high prices of basic goods are some of the major problems affecting workers both inside and outside of their working environment. Being a formal employee is no longer a guarantee for an acceptable quality of life. As a result, over 1.6 million Venezuelans have left their country since 2015 in a migration crisis never seen before in Latin America.

**Conclusion:**

Quality of life and wellbeing of most of the Venezuelan population has being deteriorated in the last 5 years and Occupational Safety and Health (OSH) is not a priority for enterprises in the middle of the economic emergency and general deterioration of daily life.

Despite the relevance of this problem, research on the subject is very limited. Recent and pertinent data is needed to properly identify and measure the risks and negative consequences that workers and families are exposed caused by the ongoing crisis.

## Introduction

As you read this paper, it is very likely that the Venezuela’s statistics have already changed as events unfold at a rapid pace.

Out of the 32 million people living in Venezuela [1], almost half are working (13,100,203 people have some sort of occupation) [2], and all of them and their family members are being affected in different ways by the current economic climate. Over the last decade, the crisis has reached its peak, bringing the lowest salaries in the world, hyperinflation levels of 130% inter-monthly [3]; severe shortages in food, medicine, auto parts and other kinds of goods. It is now common to see long lines of people, as well as “bachaqueros” [4], in the streets of Venezuela in their quest to obtain rationed and subsidized food. “Bachaqueros”, an informal worker’s phenomenon, resell subsidized products on the black market with prices up to 100 times its value, causing an incalculable loss to the treasury in tax revenues.

The declining economy and highly dangerous environments have significantly decreased the quality of life for Venezuelan workers and their families, to the point where no longer home or workplace are safe places.

Venezuela’s macroeconomic changes have negatively affected the development of this South American nation, in clear contradiction with its abundant oil reserves. Venezuela has certified 302.25 billion barrels of oil [5], thus being the most important country in the world oil context.

Venezuela possesses 24.8% of the world’s proven oil reserves [6], which makes it the country with the largest reserves on the planet. Its economy mainly depends on its revenues from the oil industry, which represent 96% of its exports volume and 11.6% of its GDP [7]. The industry is led by the huge state-oil company, “Petróleos de Venezuela” (PDVSA), which since year 2006 has led a policy of redirecting between 80 to 95% of its revenues to the public sector trough the National Fund of Development (Fondo Nacional para el Desarrollo Nacional) [8].

The historically high prices of oil since 2006 until July 2014 [9], allowed the government to increase its public expending and raise its fiscal deficit. At the same time, the government adopted policies of severe economic restrictions for the private sector, such as currency exchange controls, nationalization of private companies, land and companies’ expropriation, and price regulations, affecting a great number of workers and their families.

## Background

### Economy

#### Currency exchange controls

To understand part of the nature of the Venezuelan crisis, one must understand the complex mechanism of the existing economy and the currency exchange controls.

In Venezuela, people and organizations cannot simply go to the regular exchange market (banks, exchange bureaus, etc.) and exchange foreign currency. Instead, they must go through a bureaucratic process in an official institution [10] which instructs them to apply for limited amounts of currency needed for personal or business purposes, without any guarantee of successfully completing the process.

The latest currency exchange control was stablished in 2003, when severe political and social conflicts in the country resulted in a nation-wide strike between December 2002 and January 2003. The strike included the interruption of all productive activities in the oil industry [11]. As a result of the strike, in 2003, the country’s external revenues dropped sharply and the international reserves dropped from USD 17 MM, to USD 12 MM [12]. In response to the alarming flight of capital, it was fairly justified to introduce a currency exchange control. The crisis was quickly solved, and by the end of 2003 the international reserves reached to USD 21.4 MM (higher than before the national strike) [13]. At that moment, economists were expecting a liftoff of the currency exchange control. Such liftoff never came, and suddenly the political discourse referred to the control as “structural”, a permanent rather than temporary component of the country’s economic policy. Since the year 2004, revenues from oil exportation have exceeded previous years by far, going from an average of USD 850 per capita in 2004, to USD 3,190.6 per capita in 2008 [12]. While this flood of foreign currency continued until 2014, the exchange controls remain in place today, making the policy quite contradictory and inconsistent for several economists and the Venezuelan citizens themselves.

The United States has recently implemented important economic sanctions [14]. These sanctions will considerably reduce the income and cash flow required to finance the country’s debt commitments, which amount to over 3.5 billion dollars for the last quarter of 2017 [15]. As it has been in the past, this will likely lead to important reductions from the government in the amount of foreign currency settled for imports of goods [16].

#### Contribution of the currency exchange control to the economic crisis

Making sense of Venezuelans statistics is a challenge. The government official exchange rate (DICOM) is used by international organizations to calculate key performance indicators such as Gross Domestic Product (GDP). Many argue that the values the government provides do not reflect reality, for instance, the World Bank takes Venezuelans official GDP figure (DICOM) to rank the country, but that result is debatable to say the least. The gap between what the official rate and the non-official/off the record rate is ten-fold.

Like most of all developing oil producing countries, Venezuela suffers from the “Dutch Disease” [17], where its production system is represented mainly by a single product, in this case: oil. Taking advantage of the high profitability of the oil industry, the demand for remaining goods needed for consumption, and most economic activities are satisfied through imports, which depend on a constant flow of foreign currency to cover the acquisition of goods in the international markets.

**Figure 1:**
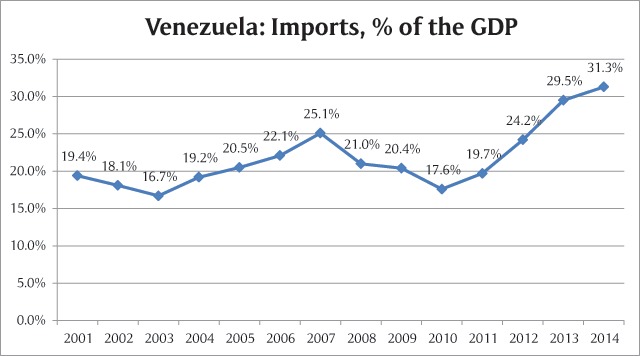
Percentage of imports in the total GDP. Data source: The World Bank 2015.

While this is an unfavorable scenario, the governmental policies have not effectively addressed the issue. Instead, import levels have increased considerably since the United Socialist Party of Venezuela (which emerged during the Bolivarian Revolution) took office in year 1999. In 2001, imports represented 19.4% of the Gross Domestic Product (GDP) and by 2014 the number increased to 31,39% (Figure 1). Official numbers for year 2015, 2016 and 2017 have not yet been published [18].

The rise on import levels translates into an increase in the general demand for foreign currency. In an exchange control system, the government is responsible for covering that need by allocating foreign currency to demanding companies or organizations. This allocation is currently done by the National Center of External Commerce (Centro Nacional de Comercio Exterior (CENCOEX)) (previously known as CADIVI), through different official exchange rates depending on the good being imported.

Since February 2004, the government has modified its control policies seven times to adjust the exchange market. However, the real problem came when oil prices suffered a substantial drop in 2014. Suddenly, the government did not have enough cash flow to satisfy the demand for foreign currency destined for import activities. According to a local financial firm, in the first 3 quarters of 2014, the CENCOEX settled a daily average of USD 79.2 million for import activities; for the same period in 2016 the number dropped to USD 12,3 million, which represents a 84.4% reduction [19]. Only pharmaceutical and basic goods companies received currency at the lowest official rate of 10 Bs/USD [20]. Venezuela will end the year 2017 with the highest inflation in the world and a severe contraction of its GDP.

In its monthly report for July 2017 “Empresas Polar”, the local leading company in the food industry, claimed it had 449 approved applications for foreign currency at the CENCOEX with a delay of 1.116 days, causing the company the loss of its credit lines with international suppliers and the accumulation of a debt over USD 130 million [21].

This situation is common amongst all production industries, leading to insufficient production levels to cover the basic needs for food, medicines and other goods of the Venezuelan people.

### Consequences

#### Inflation

Venezuela’s out of control inflation is considered the main problems affecting its people. inflation by the end of 2017 was of 2,616% [22], and is estimated to reach 1,000,000% by the end of 2018, with conditions similar to those of Germany in 1929 and Zimbabwe by the end of the 2000’s [3], making Venezuela the country with the highest inflation in the world and the most dysfunctional economy of the planet. President Maduro extended for the 14^th^ time, the 60-day decree for economic emergency after its publication for the first time in January 2016 [23,24]. Contraction of the GDP in 2017 was of –15% and the International Monetary Fund (IMF) has projected a –18% contraction of the economy for 2018 [22], adding to an accumulated –35% for the 2014–2017 period. Very few countries in the world’s history have suffered a contraction so violent that will lead to a very uncertain bleak economic scenario for 2018. The Central Bank of Venezuela (Banco Central de Venezuela (BCV)) has not published official numbers for inflation since December 2015, violating the BCV’s own regulation, which requires it to publish these indicators within the first 5 days of every new month [25].

The seven digit inflation rate and other indicators suggest hyperinflation levels [26], with prices almost doubling every month, the cost of life has become extremely challenging for the working population. Even though the government has raised the minimum wage 15 times from 2015–2018 (the most recent of 3,364.2%), the salary is equivalent to barely 30 USD, and the increases are insufficient and no match to the rampant inflation. A way to statistically measure the purchase power of the population is through the cost of the “family basic food basket”, which represents the money required to purchase the monthly goods that cover the basic nutritional requirements for a family of five members [27]. Venezuela’s National Statistics Institute, (Instituto Nacional de Estadísticas (INE)), has not published official numbers for this indicator since November 2014. However, as of July 2018, the Venezuelan Professors Association Center for Documentation and Social Analysis (CENDAS-FVM) states that the value of the family basic food basket has increased 30,385% since July 2017 [28] and it takes 98 minimum wage salaries in order to acquire it [28].

#### Industry shutdowns

The fall in foreign currency settling caused import levels to drop at alarming levels, not because the demand for foreign currency had dropped, but rather because the governmental institution (CENCOEX) has not nearly covered the demand required. Official numbers from the Venezuelan Central Bank show that imports fell 44.21%, from USD 61,591.00 million in 2013 to USD 36,591.00 million in 2014 [29]. In a live broadcast from December 2016, President Maduro announced that imports for that year had dropped to USD 17,800.00 million, a 50% decrease since its all-time high of 2014 [30]. In year 2017, different private economic and financial firms claim that imports fell around a –20%, and expect a –35% fall for 2018 [31].

The Venezuelan Confederation of Industrials (Confederación Venezolana de Industriales (CONINDUSTRIA), which represents companies from the Industrial sector, claimed that in the first quarter of 2017, manufacturers operated at less than half of their production capacity, particularly at 32.4%, a fall of 36.3% compared to the previous year. Also the average cost of production increased 731% in the first quarter of 2017 [32,33].

A survey conducted by CONINDUSTRIA in November 2017, predicts that of the 3.800 industrial companies that remain in Venezuela, 1.018 (27%) might shut their operations in 2018 mainly due the adverse macro-economic conditions and the inability to access foreign currency to acquire raw materials, machinery and equipment in international markets [34].

Some examples about the crisis are: in January 2016, the Venezuelan Association of the Plastic Industry (Asociación Venezolana de la Industria del Plástico (AVIPLA)) noted that the industry was operating at an average 50% of its installed capacity, when the plants at “El Tablazo” Petrochemical complex of PEQUIVEN (main supplier of the resins), were shut down for almost two straight months [33], which generated employers paying salaries, no production, impact on consumers

Since October 2016, “Empresas Polar” has temporarily shut down several of its production facilities 5 times, due a lack of imported raw materials (for example wheat, barley) [33], resulting in a decrease in food production.

Multinational companies that used to have their main production sites for Latin America located in Venezuela, have not escaped the problematic. Companies such as Owen – Illinois, Kimberly Clark, General Motors, Bridgestone, Clorox, Cemex, Ford Motors, General Mills and recently Kellogg’s have either been taking over by the government, permanently shut down their business in Venezuela, or temporarily paralyzed operations.

Since the year 2015, more than 14 airlines have ceased operations, leaving Venezuelans with very few remaining options to travel abroad.

Although numbers for lost jobs have not been quantified, it is estimated that more than 50.000 direct jobs and 200.000 indirect jobs have been lost by these shutdowns [35].

In 2002 there were approximately 830,000 active companies. Today, fewer than 250,000 remain operational [36].

#### Exodus

More than 1.6 million Venezuelans have left the country since 2015. For a country of over 32 million people, this represents 5% of the entire population. Every day, Thousands of people are crossing the borders hoping to reach other South American countries, engaging in journeys that may last for more than 10 days, by land transportation and recently by foot, carrying a couple suitcases with their most personal belongings. In August 2018, the International Organization for Migration (IOM), said “This is building to a crisis moment that we have seen in other parts of the world, particularly in the Mediterranean. Newspapers and NGO’s all around the world claim this is the largest migration crisis in the History of Latin America [37].

Countries like Colombia, Peru and Ecuador have declared an State of Emergency given the Influx of Venezuelan Refugees seeking to enter their territories [38]. As border control grow tighter, Venezuelans are settling in refugee camps, living in vulnerable conditions. New waves of xenophobia are spreading amongst Latin American countries.

The United Nations Refugee Agency (UNHCR) is making pressure in destination countries to protect the migrants, and has developed a regional response plan and protection guide to ensure their welfare and international assistance [39].

Migrants claim they are fleeing the country as a result of the socio-economic situation, including hyperinflation, violence, food and medicines scarcity, collapse of essential social services as well as virtually inexistent wages [39].

#### Violence

General violence could be considered as the most alarming risk factor affecting the quality of life of Venezuelans, with the soaring statistic of 1 Venezuelan dying every 18 minutes as a result of a violent event [40]. These numbers make Venezuela the country with the second highest homicide rate in the world [41].

Violence at work has been defined as any action, incident or behavior that cannot be considered a reasonable attitude and which attacks, degrades or injures a person within the scope of their work or directly because of it [42]. It can take different forms. It may be physical or psychological violence, threats or assaults, intimidation or harassment based on different grounds, including gender, race or sexual orientation [42]. In Venezuela, this behavior has become more common in certain work sectors were workers and employers could be threatened by “mafias”. An example could be the construction sector were “sindicatos” manage internal work issues [43]. In Venezuela, during the first half of 2012, the Venezuelan Observatory of Social Conflict (Observatorio Venezolano de Conflictividad Social (OVCS)) recorded 28 killings of workers and trade unionists. There are on average, five killings of trade unionists or workers per month [44]. Notably, 86% of these cases belong mainly to the construction sector, and hired assassins are among the most recurrent practices in the murders of workers and union leaders [44].

These numbers contrast with a survey by the European Union based on 15,800 interviews in its fifteen country members. Results showed that 4% of workers (6 million people) had been subjected to physical violence, 2% (3 million people) to sexual harassment, and 8% (12 million workers) to intimidation and bullying [45]. In the US, it is estimated that the total annual cost for employees due to violence in the workplace is 4,200,000 million dollars [46].

Likewise, the Venezuelan legislation conceives the concept of “*in itinere accidents*” [47]. From this definition, anything that happens in the commute could be classified as a work-related incident. However, many workers are victims of violence and attacks, but these incidents are rarely reported so there are no official statistics published, certainly leading to underreporting numbers, due to impunity. Being assaulted on public transportation or even in a private vehicle is a daily risk that an employee must face. Even simple activities such as using the phone or an electronic device could represent a serious hazard and thus hinder employees’ performance.

#### Poverty and workers

Despite Venezuela being considered a privileged and “rich in resources” country, its poverty levels in 2017 reached the figure of 87% with half of the population living under extreme poverty (61.2%) [48].

**Figure 2:**
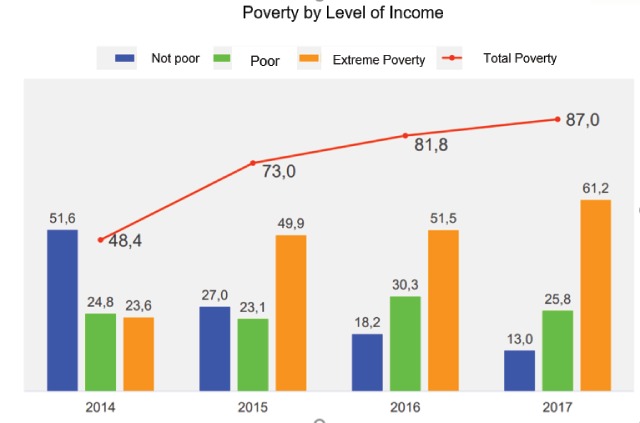
Encuesta Condiciones de Vida (ENCOVI) 2014–2017. UCAB-USB-UCV. 2014-2015-2016-2017.

It is important to emphasize that poverty has almost doubled since 2014, going from 48% to 87% [49]. Extreme poverty in 2014 was at 23.6%, and by 2017 increased to 61.25% (Figure 2).

It is noteworthy that nearly 23 million Venezuelans have trouble meeting their needs through their regular income. For the first time, extreme poverty is higher than non-extreme poverty; more than half of the country’s population does not have enough income to meet their basic food needs [49]. A study from 2016, led by the most important universities in the country, revealed that 75% of the individuals in the study referenced involuntary weight loss (9 kg in extreme cases). These results also showed that 9.6 million Venezuelans are eating twice or once per day [50].

A common indicator used worldwide for measuring poverty is the access to basic services like water, gas, electricity and telecommunications. In Venezuela, access to these services is not determined by the ability for companies and citizens to pay for them, but by the supply of these services themselves. All basic services exist at subsidized prices but offer very poor-quality service.

There have been repetitive periods of water and electricity shortages which have lasted several months, and general rationing, due to problems in the maintenance of equipment and unfavorable weather conditions. Several industries have reported important deficits in their production due electricity blackouts and a lack of water supply. Internet and telephone services in Venezuela have been ranked as the worst in all Latin America. Many multinationals companies rationalize the poor-quality service as the result of the fixed prices for the telecom industries, and many of them have expressed the intention to cease their operations in Venezuela. It is fairly common that companies lose an entire working day from internet connectivity problems [51].

#### Scarcity of basic goods

The Pharmaceutical Federation of Venezuela (Federación Farmacéutica Venezolana) stated that by January 2017, the shortage of medicines reached 85% [52], and the shortage of basic products, according to extra-official sources reached 80% in June 2016 [53]. As a consequence, people spend up to 3 hours in line to access a limited number of products, which sometimes do not meet the needs of their families [54]. A new dramatic issue in Venezuela is that many workers use up most of their free time trying to buy essential goods generating frustration, since the citizens cannot spend that time with their families and children and participate in fun or recreational activities [55]. It is very common that workers are absent from work to obtain rationed food and medicines, since there is a single day per week for purchasing these goods, depending on their ID number and customers are fingerprinted.

#### Health impact

Venezuelan health system is collapsing [56]. If health is defined by the World Health Organization (WHO), as the a state of complete physical, mental, and social well-being and not merely the absence of disease or infirmity [57], anyone living in Venezuela could not be completely healthy having to comply with the scarcity of basic products and goods, limited access to medication and health care, low purchasing power, violence, instability, inflation, and uncertainty.

The economic crisis described has sparked a rapid deterioration of the Venezuelan health system. In early 2017, the former Minister of health retroactively published the bulletins corresponding to 2015 and 2016, which documented the alarming deterioration in the overall health status of the population (e.g. a 30% increase in mortality among children under one year of age). After this information was published, the Minister was fired [58]. Hospitals and Medical Schools have been vandalized, equipment has been stolen or is in deplorable shape, and syringes and other surgical material are being re-used [58] affecting not only patients but health care workers. To make matters worse, there is 85% scarcity of medication at local pharmacies [40]

In Venezuela, according to data collected from 2004 through 2005, every day, an average of 4 people lost their lives as a result of accidents in their jobs [41]. The number of accidents has a rate of thirty-three [33] accidents per hour; injured workers exceed this number, and the number of disabled employees exceeds 18.4% [59].

Between 2002 and 2006, the Institute of Prevention, Health and Safety (Instituto Nacional de Prevención, Salud y Seguridad Laborales (INPSASEL) reported 1,011 occupational illnesses, and in 2006 the number increased to 2,066 which represents an increase of 78.56% [60], maybe due to initial underreporting when the institute was created. Considering the statistics published by INPSASEL in relation to work related accidents in Venezuela, 34,202 accidents were registered in 2006, and 52,458 accidents in 2014 [61].

Venezuela is experiencing an acute public health emergency with a breakdown of the medical infrastructure [62]. There are shortages of food, water, electricity, medicine, and medical supplies that have contributed to an increasing humanitarian crisis affecting much of the country: The country is experiencing outbreaks of infectious diseases: There has been a 205% increase in new Malaria cases [58] In 2017, over 400,000 cases of Malaria were reported [62]; there is almost a complete lack of access to tuberculosis screening for vulnerable populations such as prisoners and indigenous communities [58] and there are no official numbers about other mosquito borne illnesses (Dengue, Zica, Chicungunya and yellow fever). All these reasons have led CDC to publish a warning –level 3, avoid nonessential travel to Venezuela.

#### Psychosocial factors and wellbeing

The World Health Organization (WHO) defines psychosocial factors at work as the interactions between work, the environment, job satisfaction and conditions of the organization. The worker’s abilities, their needs, culture and personal situation outside of work, all of which, through perceptions and experiences, may influence health and job performance and satisfaction [63]. The broad concept of psychosocial factors at work gives us the perspective on why the Venezuelan crisis is directly affecting the everyday working life.

**Figure 3:**
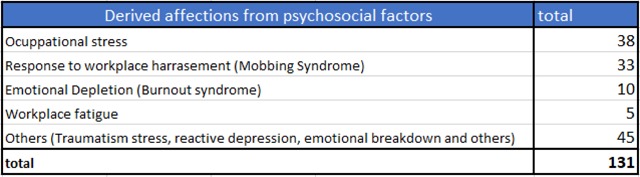
Number of reported occupational diseases derived from psychosocial factors in 2006. Source: Epidemiology Department of the National Institute of Prevention, Health and Social Security, 2006.

In Venezuela the list of occupational diseases includes the conditions resulting from psychosocial factors (occupational stress, burnout and response to workplace harassment (Mobbing syndrome)). The Department of Epidemiology at the National Institute of Prevention, Health and Safety (INPSASEL) stated in its 2006 report of occupational diseases that the conditions caused by psychosocial factors were the second major health problem diagnosed in the regional agencies of workers health services, with 131 cases, which constitute 6.3% of the total occupational diseases that were diagnosed in this institution [64] (Figure 3). However, there are no reports of the chronic stress Venezuelan citizens are going through.

Although in the list of medically coded occupational diseases (ICD–10), the terms “stress” and “burnout” are not directly referenced, they could be interpreted within the classification “Other mental or behavioral disorders”, when a link is established between exposure to risk factors arising from work activities and the mental disorder contracted by the worker [65]. However, in Venezuela there are not enough qualified psychologists to diagnose and stablish the link between work and stress, which certainly present, especially during civil unrest. The Venezuelan Observatory of Social Conflict (SVCO) recorded at least 2,836 protests for the first half of 2015, which represents an average of 16 daily protests across the country. Similarly, robberies, murders, abductions and political tension have resulted from the economic, social, political and moral crisis of the Bolivarian Republic of Venezuela.

From April to August 2017, a wave of anti-government protest erupted with great violence. A total of 6,729 protest were registered. 163 people died while protesting [66]. Barricades were placed across the main roads of the cities preventing access to the workplace by workers. Sudden evacuations due to violence on the streets, numerous lootings and brief nationwide strikes affected the regular operations of most industries and commerce in the country. No studies have yet been published on this subject, although general stress, violence, anguish, and other psychological effects affecting mental health, general wellbeing and creating absenteeism were evidently felt across the country.

#### Climate Change and impact in worker’s life

Venezuela does not escape from the alert on the negative effects workers must deal these days, as a result of climate change [67]. These can be summed up in: Changes in morbidity and mortality from temperature changes, health impacts from extreme meteorological events (hurricanes, storms, tornados, high precipitations and droughts). In Venezuela, 70% complained of intermittent water supply [56]; other issues not well documented are: a) air pollution and an increase of its related health effects, b) reduction of the output from the agriculture and farming industries, c) diseases transmitted from polluted water and food, d) and other infectious vectors.

In a recent study on *The impact of high emissions of CO*_2_ *and general pollution*, from the WHO, it was confirmed that 92% of the world population, lives and works in places were air pollution exceeds the level considered as “permissible” [68]. In Venezuela statistics are not available but it is known that ecological measures are not a priority regarding general pollution.

In Venezuela, many workers move daily from peripheral cities to their workplace in transportation that does not have any environmental regulation, thus leaving a much greater carbon footprint for the use of 2 or 3 combustion vehicles.

In recent years, urbanism projects in Venezuela amounted to the construction of 1.4 million houses according to government figures. Likewise, the exponential growth of informal settlements has reached staggering levels. This type of construction has been considered the “main cause of pollution on the planet” according to experts of the Forum of Santiago, who explain that in these settlements, proper management of solids waste is not achieved, causing pollution in the water, soil and air, along with the social impact implicit in this disorder. The Green City Index of Latin America Report states: “The informal settlements are, by definition, unsustainable. They represent a high degree of social and economic exclusion”. One of the most advanced Latin American thinkers, Milton Santos, quoted: “poverty is the worst form of pollution.” [69,70] and taking in consideration that 81,77% of the population in Venezuela live under poor conditions it could be inferred the ecological consequences that this entails.

#### Leisure time deterioration

The social dynamic has changed in the last 10 years, due to many factors. When we address leisure time, we refer to time free of obligations of any kind; it is the privilege of dealing with pleasant and useful things to one’s own desire, whether for resting, amusement or personal development [71]. In general, leisure time has been reduced for the average Venezuelan families as well as “recreation”, which is framed as those opportunities for the use of free time offered by society, which enable leisure experiences [72].

In this regard, the development of life with increasingly long periods for enjoyment, generates and promotes an essential culture of wellness, through recreation and social tourism [72]. Having mentioned this limitation, one can expect a deterioration on the general wellbeing of the workers and their extended families, taking into consideration that physical and recreational activities are vital to the development of the human being, who by nature requires an outdoor setting, where it can act out its aspirations and concerns [73]. It is noteworthy that in Venezuela, the right to sport and recreation has a constitutional degree; article 111 of the Constitution of the Bolivarian Republic of Venezuela, states that everyone has the right to sport and recreation, as activities that benefit the quality of individual and collective life. The State assumes responsibility for sports and recreation as an educational and public health policy, and must ensure the resources for its promotion [71]. In addition, Article 55 of the LOPCYMAT, states that workers are required to comply with health, safety, ergonomics, and prevention policies; and to participate in programs for recreation, use of leisure, rest and social tourism that will improve their quality of life, health and productivity [47].

The draft for an Organic Law for Recreation establishes the need to regulate the protection, promotion, organization, planning, coordination and execution of public policies on recreation, as a right which guarantees the full development of human potential, its personal, social and community growth [47]; The Organic Law for Tourism, in its 57^th^ article, first paragraph, states that “…it is a state policy for ensuring those living in the country, the access to the exercise of the right to rest, recreation and use of leisure time, in appropriate conditions of safety and comfort …” [74].

While it is true that in Venezuela’s government has a good legal base, policies, laws and regulations (and even a Vice Minister for Supreme Happiness of the people), which should protect wellbeing in general of the Venezuelan citizens, these measures have failed to materialize. The written regulations are a big contrast with the reality that affects leisure time and recreation for the Venezuelan workers. Many workers need 2 or more jobs and need increasing amounts of time to search basic goods. The subsistence-level wages and street violence, difficulties in the commute (deficiency in public transport services, high costs of space parts for private cars, taxis), severely limit the access to outdoors activities, meals in restaurants, movies, night club activities, walking freely on streets, exercise, or even travel inside the country for touristic purposes.

## Conclusions

Life in the big cities of Venezuela is characterized by a pattern of constant stress. During a disorganized environment, there are double workloads, low purchasing power, product shortages, above par conditions of basic services (water, electricity, internet connection), poor mobility, pollution, poverty, street violence and even violence in the workplace or during the daily commute are variables that create stress and affect the Venezuelan workers’ wellbeing in general. Macroeconomic factors are severely affecting the worker’s quality of life. The lack of job opportunities, low salaries, and scarcity levels have led to an increase of the informal economy sector. Many workers seek more than one job to “survive”. Half of the country’s population is now in the “Extremely-poor” bracket.

The Venezuelan migration crisis is seen now as the worst in Latin America’s history, with thousands of Venezuelans currently under refugee status, living in extremely vulnerable conditions.

Almost all industries have reported temporary and permanent shutdowns of production life, lack of raw materials and forced staff reduction. The number of active companies has gone down dramatically in the last decade, thus decreasing the formal job opportunities and the number of formal workers with proper OSH working conditions. Work incidents almost double in the 2006–2014 period, even though Venezuela has one of the world’s best legislations in respect to occupational safety and health.

There is a paradox that Venezuela has a Vice Ministry of Supreme Social Happiness for the People [75], and at the same time, Venezuela’s ranking on the world happiness index [76] has plummeted in the last five years. There are many factors that play part on people’s happiness, and all the metrics shown before depict how Venezuelan’s quality of life is steadily deteriorating.

On the positive side, Venezuelans are exposed to constant opportunities to build resiliency which has to be a component of wellbeing for those living in the country [77]. Even though there is no objective data, yoga practice and meditation have become more popular in the country, increasing solidarity behavior and learning to overcome difficulties. For the poor sector, the time used on recreation, now is spent in waiting in lines for cash, rationed goods and medicines or finding “an additional way to survive”. The growth of slums in the main cities has a major impact on environmental conditions and pollution were workers live.

The political and socio-economic situation has led to a mass exodus of more than 1.6 million highly qualified and talented professionals, and a total of more than 2.5 million of Venezuelans leaving the country [41]. This number seems to grow daily.

Despite the relevance of this problem, research on the subject is very limited. Official numbers and statistics are practically non-existent since the year 2014.

Recent and pertinent data is needed to properly identify and measure the risks at work caused by the ongoing crisis, and the negative health consequences of the current working and living conditions for the working people of Venezuela.

Venezuela’s crisis is the result of a long process of political unrest and questionable bad economic decisions [58]. As long as the crisis in Venezuela continues and the civil society in the country continue to respond, with minimal resources, OSH will be a luxury rather than an obligation for companies and organizations [78]. There are few objective data about the negative impact of the unprecedented crisis, since research is not sponsored and has declined in the latest years [79], however looking back at the scenario it could be inferred that if the situation does not change all the indicators will be worse in the future with severe implications for the future generations. If remedial political and economic decisions are not taken soon, how then will the working conditions for the Venezuelan citizens be from the biopsychosocial perspective in the next 5 years?
